# Necrotizing Fasciitis of the lower extremity: a case report and current concept of diagnosis and management

**DOI:** 10.1186/1757-7241-17-28

**Published:** 2009-06-15

**Authors:** GA Naqvi, SA Malik, W Jan

**Affiliations:** 1 Department of Orthopaedics, Our Lady of Lourdes Hospital, Drogheda, Republic of Ireland

## Abstract

Necrotizing fasciitis is a severe soft tissue infection characterized by rapidly progressing necrosis, involving subcutaneous tissues. This rare condition carries high mortality rate and require prompt diagnosis and urgent treatment with radical debridement and antibiotics.

We describe a case of 21-year old man who presented with the history of trivial injury to the knee. Initially he was admitted and treated for septic arthritis but later was diagnosed as necrotizing fasciitis which was successfully treated with no ill effects what so ever from this devastating condition.

This rare condition has been reported in literature but still early diagnosis, which is a key for successful treatment, remains a challenge.

## Background

Necrotizing fasciitis(NF) is a severe soft tissue infection characterized by rapidly progressing necrosis involving mainly the fascia and subcutaneous tissues, but can also extend to involve muscles and skin. This rare, life-threatening condition has been recognized since 18^th ^century with various names including phagedena gangrenosum, hospital gangrene, Meleney's gangrene, Fournier's gangrene etc. Although rare, it is frequent enough that surgeons will likely have to be involved with the management of at least 1 patient with NF during their practice, but it is infrequent enough to achieve complete familiarity with the disease. Establishing the diagnosis of NF can be challenging in treating these patients, and knowledge of all available tools is key for early and accurate diagnosis. The purpose of this article is to review the different tools available for diagnosis and the treatment principles for NF.

## Case

A 21-year old man with no co-morbidities was referred to the regional orthopedic unit from emergency department of another hospital in the vicinity, with the history of trivial injury to his right knee two days ago. He accidentally hit his right knee to the wall two days back and sustained an abrasion to his knee. He started complaining of pain in his knee the next day and had to stop working. Pain got worse over night and he attended the emergency department the next day, from where he was referred to us with the suspicion of septic arthritis or cellulitis. He received intra-venous benzyl-penicillin and flucloxacillin in the emergency department.

On arrival, he was afebrile and systemically stable but in considerable pain. Examination of the right knee revealed small superficial wound over patella, slight redness and increased temperature in surrounding area with grade I effusion in the joint. Movements of the joint was reduced and associated with severe pain. Neurological and vascular examination of the limb was satisfactory.

The initial blood investigation revealed white cell count (WCC) count of 18.6 × 10^9^/L, C-reactive protein (CRP) of 63.1 mg/L, and hemoglobin (Hb) level of 15 g/dL. X ray of the right knee did not show any bony injury or gas in the soft tissues. A working diagnosis of septic arthritis secondary to traumatic wound was made and urgent arthroscopic washout of the knee was performed that night. Arthroscopy revealed inflamed synovium and only 10 cc of fluid was drained from the knee. Urgent microscopy and gram stain of the fluid and later culture failed to reveal any organism.

**Figure 1:**
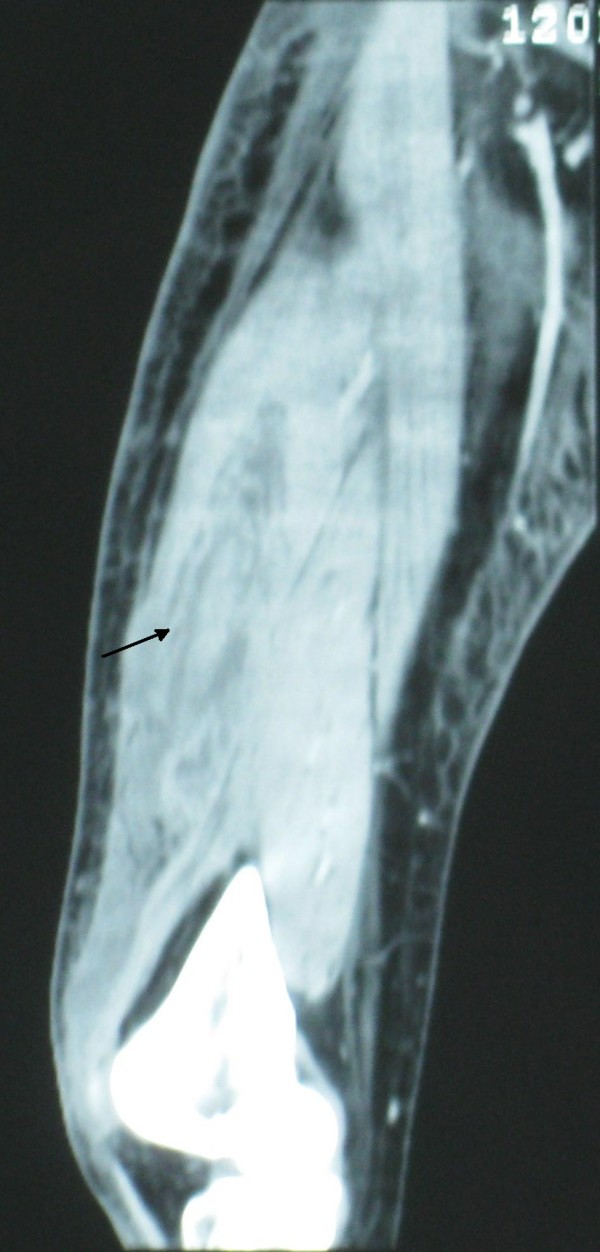
**CT scan of right thigh, showing inflammatory stranding and low attenuation in vastus latralis (arrow)**.

**Figure 2:**
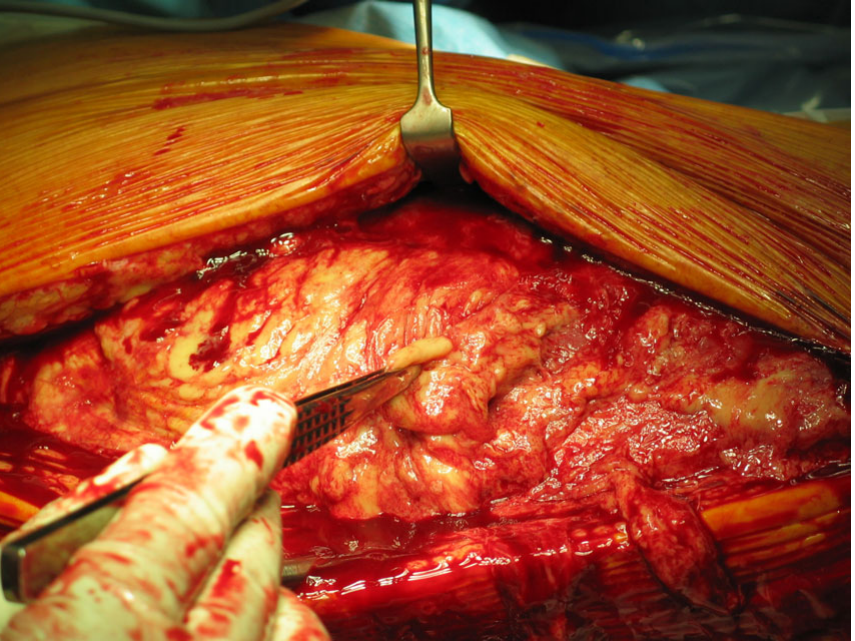
**Intra-operative picture of surgical debridement of thigh through antero-lateral approach**.

**Figure 3:**
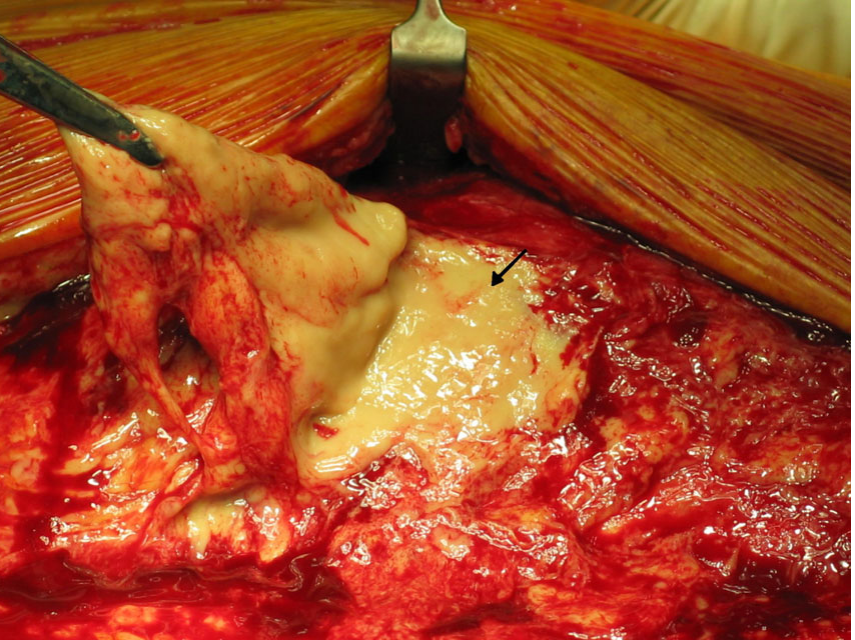
**Intra-operative picture showing necrotic fascia and subcutaneous tissue as evident by lack of bleeding (arrow)**.

Post-operatively, intravenous flucloxacillin and benzyl penicillin was continued along with gentamycin. Even after 72 hours of antibiotics patient remained symptomatic. Although he remained afebrile at all times, his pain and tenderness continued to increase in distal thigh and blood investigations revealed a marked increase in inflammatory markers (CRP of 181 and ESR of 37). Considering the failure to respond with intravenous antibiotics, negative arthroscopy and increasing tenderness in distal thigh, a suspicion of necrotizing fasciitis was made. Urgent contrast enhanced Computed Tomogram (CT) scan (Fig 1) of his right thigh and knee was performed that revealed marked inflammatory stranding and low attenuation with suspicion of necrosis mainly in rectus femoris and vastus lateralis. Patient was taken to theatre urgently and fasciotomy performed through antero-lateral approach of the thigh which confirmed the CT findings of necrotic fascia and muscles (Fig 2 and 3). Thorough debridement of rectus femoris and vastus lateralis was performed, wound was washed thoroughly and packed with betadine soaked swabs.

The case was discussed with microbiologist and intra-venous (IV) clindamycin and ciprofloxacin was added along with flucloxacillin and benzyl penicillin. IV hydration and oxygen therapy was maintained through out, with close observation of renal functions which remained stable.

Patient was taken to theatre again in 48 hours for re examination which showed more necrotic area in rectus muscle which was debrided again. Further two washouts with 48 hours interval, did not show any progression of necrosis and wound was closed gradually with staples and shoe lace technique. IV antibiotics were continued for 14 days followed by oral clindamycin and ciprofloxacin for 5 weeks. He remained stable systemically and responded well to the treatment as evidenced by normalizing inflammatory markers. The diagnosis was confirmed on tissue histology but causative organism remained unidentified.

Patient made an amazing recovery from this limb and life threatening condition which was made possible by multi-disciplinary approach involving orthopedics, general surgery, radiology, microbiology, physiotherapy and dieticians. Patient was discharged home after 19 days of in-hospital stay. At final follow up 3 months later he had full range of motion in his right knee and grade 4 power in knee extensors.

## Disscussion

Necrotizing fasciitis is a rare, potentially fatal bacterial infection characterized by widespread necrosis of the subcutaneous tissue, superficial fascia and skin. This rare, life-threatening condition has been recognized since 18^th ^century with various names including phagedena gangrenosum, hospital gangrene, Meleney's gangrene, Fournier's gangrene etc. A Confederate Army Surgeon, Joseph Jones, wrote one of the earliest descriptions of necrotizing soft tissue infections in soldiers during the American civil war in 1871 and reported a mortality rate of 46% [1]. In 1883, the French physician, Jean Alfred Fournier, described a similar NSTI of the perineum in five male patients – a process that continues to bear his name. In 1952 the condition was described as necrotizing fasciitis by Wilson for the first time to include both gas-forming and non-gas-forming necrotizing infection and stated that fascial necrosis is the sine qua non of this process. [2]. More recently, the term necrotizing soft tissue infection (NSTI) has been suggested to encompass all of these necrotizing infections and advocate an approach to all of them that uses the same principles for diagnostic and treatment strategies. This will allow for earlier diagnosis and expedited treatment, which are essential for improving outcomes and decreasing mortality in patients with NSTI [3].

### Incidence and classification

The incidence has been reported approximately 1,000 cases per year in the United States or 0.4 to 0.53 cases per 100,000[4,5]. Not only individuals with pre-existing co-morbidities are affected but also young and healthy individuals can be affected. NSTI can be classified according to the anatomic location involved or the depth of infection as necrotizing adiposities, fasciitis, or myositis. Most commonly NSTI is classified on the basis of microbiology as type I, being polymicrobial, type II, being monomicrobial and type III, being caused by marine vibrio species. These classification systems are not clinically significant as they do not change the management of the patient. This condition can involve any part of body but primarily involves extremities, abdomen or perineum. Anaya et al in their study of 150 established cases of necrotizing fasciitis estimated that extremities were the most common site of infection in 57.8% followed by the abdomen and perineum [6].

### Microbiology

There are two main groups of necrotizing fasciitis depending on microbiology. Type-I NF are polymicrobial. Approximately 55% to 75% of all cases result from type I infection and most common bacterial species include Gram-positive cocci (streptococci, staphylococcal species), enterococci and gram-negative enterobacteriaceae (Escherichia coli, Acinetobecter species, Pseudomonas species and Klebsiella species). Bacteroides species are the most common anaerobes, while Clostridial species are an infrequent isolate [7-11]. Type I infections are often diagnosed in immunocompromised patients and tend to occur in the perineal and trunk areas [12]. Type-II infections are monomicrobial and usually caused by group A Streptococcus (Streptococcus pyogenes) either alone or in association with Staphylococcus aureus. Streptococal infection can be associated with toxic shock syndrome. Recently there has been an increasing incidence of community-acquired methicillin-resistant Staphylococcus aureus (MRSA) soft-tissue infection, particularly in IV drug abusers [13-15]. Some other organisms has been reported as a rare cause of type-II infection like Klebsiella species [16] and group-B streptococcus [17]. Type II NF is far less common than type I infection and tends to occur in otherwise healthy, young, immunocompetent hosts and is classically located on the extremities. There is another subtype due to marine Vibrio species (e.g. V. vulnificus) [18] associated with marine injuries and contact with raw sea food. Although this is the least common type, it is associated with a fulminant course and can lead to multisystem organ failure within 24 hours of infection. Wong et al [10] in their study of 89 patients with necrotizing fasciitis described the causative micro organisms as obtained from first debridement sample. According to their study a single organism was identified in twenty-five patients (28.1%), multiple organisms were identified in forty-eight patients (53.9%), and no organism was identified in sixteen patients (18.0%). Streptococcal species, identified in thirty-one patients, were the most common isolates. Forty-eight (66%) of the positive cultures were polymicrobial necrotizing infections (Type-I NF).

### Pathophysiology

Necrotizing fasciitis is characterized by rapidly spreading infection in the subcutaneous tissues. Microbial invasion of the subcutaneous (SC) tissues occurs either through external trauma or direct spread from a perforated viscus (particularly colon, rectum, or anus). Bacteria then track SC, producing endo and exotoxins [19] that cause micro-vascular thrombosis[20], tissue ischemia, liquefactive necrosis, and often systemic illness [21] which can progress to septic shock, multisystem organ dysfunction, and death. Tissue ischemia, impedes oxidative destruction of bacteria by polymorphonuclear cells and prevents adequate delivery of antibiotics. Hence, surgical debridement is the mainstay therapy for NSTI, and antibiotic therapy alone is of little value and only mask the severity of the symptoms [22].

### Risk factors

The commonest predisposing factor for NF of extremity is a history of drug abuse and multiple needle punctures in the affected site, this was found in 129 (33%) patients in a systematic review of NF of upper and lower limb[23]. Other predisposing factors include trauma, fish-fin injury, chronic skin ulcer, burns, post operative wound infection, insect bite and colo-cutaneous fistula [24-27].

Diabetes Mellitus(DM) is the leading predisposing underlying medical condition because of peripheral neuropathy, peripheral vascular disease and immuno-suppression. According to Elliot et al [7] presence of DM does not affect mortality unless it occur with certain other diseases. Other associated diseases include immuno-deficiency, alcoholism, chronic renal failure, liver cirrhosis, HIV, malignancy, steroids and peripheral vascular disease [28-30].

### Clinical manifestations

Lack of cutaneous findings early in the disease make the diagnosis challenging and high index of suspicion is essential. Common sign and symptoms of the disease are summarized in [Table 1]. The most common early signs are erythema, local warmth, skin induration, and edema. These signs often make early diagnosis difficult and the condition is often diagnosed as cellulites, abscess or septic arthritis as in our case and the diagnosis of necrotizing fasciitis is only suspected when patient fail to respond to broad spectrum intravenous antibiotics or develop cutaneous manifestations. Pain out of proportion to the apparent severity of the lesion should alert the physician to the possible diagnosis of Necrotizing fasciitis. Patches of skin necrosis, tissue crepitus, fluctuance and systemic evidence of sepsis such as hyperthermia, tachycardia and hypotension are alarming signs.

**Table 1:** Clinical manifestations of Necrotizing Fasciitis

Erythema
Pain
Warmth
Edema
Induration
Fluctuance
Crepitus
Skin necrosis
Bullae
Abscess
Fever
Hypotention

### Diagnostic tools

Early diagnosis of NF is not always possible due to paucity of cutaneous findings early in the disease therefore high index of suspicion is important in infected cases that are refractory to antibiotics. There are a wide variety of diagnostic tools that can be used as an adjunct for diagnosis.

Wong et al. [31] created a score (laboratory risk indicator for necrotizing fasciitis score) to discriminate between NSTI and non-necrotizing soft-tissue infection. They identified 6 independent variables associated with NSTI. Each variable, if present, gives a specific number of points toward the final score [Table 2]. The total score had a range of 0–13, and Wong and colleagues showed that, for intermediate and high-risk patients (score, > 6) had a PPV of 92% and a NPV of 96% [31].

**Table 2:** Laboratory Risk Indicator for Necrotizing Fasciitis Score [31].

Variable	Score
C-reactive protein	
	
< 150	0
≥ 150	4
	
WBC(cells/mm^3^)	
	
< 15	0
15–25	1
> 25	2
	
Hemoglobin(g/dL)	
	
> 13.5	0
11–13.5	1
< 11	2
	
Sodium(mmol/L)	
	
≥ 135	0
< 135	2
	
Creatinine(mcg/L)	
	
≤ 141	0
> 141	2
	
Glucose(mmol/L)	
	
≤ 10	0
> 1	1

Other diagnostic adjuncts like ultrasound, CT scan and magnetic resonance imaging (MRI) are helpful in suspicious cases. The usefulness of MRI in the diagnosis of NF has been supported in a study by Rahmouni et al. [32], who in 36 patients were able to differentiate between non-necrotizing cellulites that would respond to medical treatment and severe necrotizing infection that require rapid, life-saving surgery. Wang and Hung [33] investigated the use of tissue oxygen monitoring with near-infrared spectroscopy for the diagnosis of NF. They reported that tissue oxygen saturation less than 70% had a sensitivity of 100% and a specificity of 97%. The authors of this study propose that this method may offer a reliable non-invasive approach of assessing lower extremities at risk of necrotising fasciitis. Examination of a frozen section biopsy specimen from the compromised site that includes deep fascia and possibly muscle has been suggested as well, as a means to achieve earlier diagnosis of NSTI in patients [20,34].

### Treatment

Early diagnosis and prompt treatment including surgical debridement and braod spectrum antibiotics is the key to successful treatment in necrotizing fasciitis [35,36]. The initial regimen should include agents effective against aerobic gram-positive cocci, gram-negative rods, and a variety of anaerobes [37]. The usual multidrug regimens include high-dose penicillin, high-dose clindamycin, and a fluoroquinolone or an aminoglycoside for coverage of gram-negative organisms. Vancomycin, or linezolid should be considered until MRSA infection has been excluded [3]. This should be accompanied with supportive measures such as fluid replacement, blood pressure support, analgesia, nutritional support and intensive care involvement etc. Intravenous immunoglobulins (IVIg) [38,39] and Hyberbaric oxygen (HBO) therapy has also been suggested as an adjunct to other treatments. There is no agreement as to the usefulness of HBO for NF [40,41].

When NF is established or highly suspected, urgent exploration and debridement of tissue is the cornerstone of successful management [37]. Surgical exploration also aid in the diagnosis of NF. The operative findings of grayish necrotic deep fascia, a lack of resistance to blunt dissection, lack of bleeding of the fascia, and presence of foul-smelling "dishwater" pus confirms the diagnosis. Tissue samples should be sent for gram staining, culture and histology. C H Wong [42], described his approach to debridement in NF which consists of 4 steps: (1) confirming the diagnosis (2) defining the extent of fasciitis; (3) surgical excision; and (4) post-excision wound care. The extent of the infection is defined by probing the wound bluntly, followed by systematic excision. He classified the infected skin into zones 1, 2, and 3. Zone 1 is necrotic tissue. Zone 2 is infected but potentially salvageable soft tissue, and zone 3 is non-infected skin. Zone 1 is completely excised. Zone 2 is meticulously assessed and cut back as necessary to remove nonviable tissue while maximally preserving salvageable tissue. Zone 3 is left alone.

The aim of surgical debridement is to remove all infected tissue in a single operation. This halts the progression of the fasciitis and minimizes unnecessary returns to the operating room. In most of the cases repeat debridement in needed before wound closure.

Wound closure should be carefully planned as early closure carries the risk of residual infection and poor wound healing. The wound must demonstrate that the healing phase has started before any attempt to closure. Several methods has been used for wound closure such as secondary suturing, shoe lace technique, skin grafting, muscle flaps etc. Vacuum-assisted closure (VAC) [24] technique has found an extended use in the field of open wound management and has shown decreased morbidity associated with wound care.

### Mortality

The mortality associated with the disease is high and has been reported from 6% to as high as 76% [43-51] [Table 3]. McHenry et al [9] in their study reviewed the determinants of mortality in patients with necrotizing soft tissue infections. They summarized the reported mortality rates from different studies. The cumulative mortality rate was 34% (6% to 76%) and 29% in their series of patients. The average time from admission to operation was 90 hours in non-survivors versus 25 hours in survivors. They concluded that early debridement was associated with a significant decrease in mortality. Similar findings were also reported by Wong et al [10]. In a retrospective review of 89 consecutive patients with necrotizing fasciitis, they concluded that the most common associated co-morbidity was diabetes mellitus (sixty-three patients; 70.8%). Advanced age, two or more associated co-morbidities, and a delay in surgery of more than twenty-four hours adversely affected the outcome. Multivariate analysis showed that only a delay in surgery of more than twenty-four hours was correlated with increased mortality [10].

**Table 3:** Reported mortality rates from Necrotizing Fasciitis.

Authors	Year	No. of cases	No. of Mortalities	Percentage
Meleney [43]	1924	20	5	20
Wilson [2]	1952	23	2	9
Rea and Wyrick [44]	1970	44	13	30
Stone and Martin [45]	1972	63	48	76
Rouse et al [46]	1982	27	20	73
Majeski and Alexander [36]	1983	30	10	33
Pesa and Howard [47]	1985	33	11	33
Sudarsky et al [48]	1987	33	2	6
Clayton et al [49]	1990	57	10	18
Francis et al [50]	1993	25	6	24
Brown et al [51]	1994	54	19	35

## Conclusion

NF is a rare life threatening condition that requires prompt recognition and aggressive surgical debridement along with broad spectrum antibiotics. Early diagnosis of the condition poses a challenge and high index of suspicion in crucial. Surgical debridement must be aggressive and complete and new therapeutic modalities like HBO and VAC may be considered for wound management.

## Consent

Written informed consent was obtained from the patient for publication of this case report and any accompanying images. A copy of the written consent is available for review by the Editor-in-Chief of this journal.

## Competing interests

The authors declare that they have no competing interests.

## Authors' contributions

GN and SM have been involved in drafting of this manuscript and WJ has revised and corrected the manuscript and given final approval for the publication. All authors read and approved the final manuscript.
